# Implementing a seventh-order linear multistep method in a predictor-corrector mode or block mode: which is more efficient for the general second order initial value problem

**DOI:** 10.1186/2193-1801-3-447

**Published:** 2014-08-20

**Authors:** Samuel N Jator, Leong Lee

**Affiliations:** Department of Mathematics and Statistics, Austin Peay State University, Clarksville, TN 37044 USA; Department of Computer Science and Information Technology, Austin Peay State University, Clarksville, TN 37044 USA

**Keywords:** General second order; Initial value problems; Block form

## Abstract

**Abstract:**

A Seventh-Order Linear Multistep Method (SOLMM) is developed and implemented in both predictor-corrector mode and block mode. The two approaches are compared by measuring their total number of function evaluations and CPU times. The stability property of the method is examined. This SOLMM is also compared with existing methods in the literature using standard numerical examples.

**AMS Subject Classification:**

65L05; 65L06

## Introduction

Linear multistep methods (LMMs) of the form
1

have been extensively applied to solve the special second order initial value problem (IVP)
2

on the discrete set of points  (see Lambert and Watson (1976), Ramos and Vigo-Aguiar (2005), Ixaru and Berghe (2004). Despite the successful application of (1) to solving problems of the form (2), fewer methods of the form (1) have been proposed for solving the general second order IVP
3

Some of the methods available for directly solving (3) are due to Awoyemi (2001) and Ramos and Vigo-Aguiar (2006). These methods are generally implemented in a step-by-step fashion in a predictor-corrector mode.

In this paper, we construct the continuous form of (1) which has ability to generate several methods which are combined and implemented in block form to solve (3) directly (see Jator and Li (2009) and Jator (2012, 2010, 2007).

The paper is organized as follows. In Section ‘SOLMM’, we derive a continuous approximation which is used to obtain the discrete methods that are combined to form the block method. The analysis and computational aspects of the SOLMM is given in Section ‘Implementation of the SOLMM’. Numerical examples are given in Section ‘Numerical examples’ to show the accuracy and efficiency of the method. Finally, the conclusion of the paper is discussed in Section ‘Conclusion’.

## SOLMM

### Continuous form

On interval *t*_*n*_≤*t*≤*t*_*n* + 6_, the exact solution to (3) is approximated by the continuous form of the SOLMM
4

whose first derivative is given by
5

where *α*_0_(*t*), *α*_1_(*t*), and *β*_*j*_(*t*), *j* = 0,1,2 are continuous coefficients that are uniquely determined. We assume that *y*_*n* + *j*_ is the numerical approximation to the analytical solution *y*(*t*_*n* + *j*_), *yn* + *j*′ is an approximation to *y*^′^(*t*_*n* + *j*_), and *f*_*n* + *j*_ = *f*(*t*_*n* + *j*_,*y*_*n* + *j*_,*yn* + *j*′), *j* = 0,1,…,6 is supplied by the differential equation. The coefficients of the method (4) are specified by the following theorem.

#### **Theorem1**

In order to obtain the coefficients of the continuous method (4), a nine by nine system is solved with the aid of Mathematica by demanding that the following conditions are satisfied


After some algebraic manipulations, the equivalent form (6) produces the coefficients of (4) whose first derivative is given by (7),
67

where we define the matrix *W* as


and *W*_*j*_ is obtained by replacing the *j*^*th*^ column of *W* by *V*; *P*_*j*_(*t*) = *t*^*j*^,*j* = 0,…,8 are basis functions, and *V* is a vector given by *V* = (*y*_*n*_,*y*_*n* + 1_,*f*_*n*_,*f*_*n* + 1_,…,*f*_*n* + 6_)^*T*^. We note that *T* is the transpose.

#### Proof

See Jator (2012).

### Discrete by-products

The following methods which are used to construct the block form are obtained by evaluating (4) and (5) at *t* = {*t*_*n* + 2_,*t*_*n* + 3_,*t*_*n* + 4_,*t*_*n* + 5_,*t*_*n* + 6_} and *t* = {*t*_*n*_,*t*_*n* + 1_,*t*_*n* + 2_,*t*_*n* + 3_,*t*_*n* + 4_,*t*_*n* + 5_,*t*_*n* + 6_} respectively.
8

The derivatives are given by
9

### Block form

The methods (8) and (9) are combined and expressed in the form
10

where


and *A*_0_, *A*_1_, *B*_0_, and *B*_1_ are matrices of dimension 12 whose entries denoted by *α*_*j*_ = *α*_*i*,*j*_, *β*_*j*_ = *β*_*i*,*j*_,*i* = 1,…,12 are given by the coefficients of (8) and (9).

### Order and local truncation error

Define the local truncation error of (10) as
11

where


and Ł[ *z*(*t*);*h*] = (Ł_1_[ *z*(*t*);*h*],…,Ł_6_[ *z*(*t*);*h*],Ł_7_[*hz*^′^(*t*);*h*],…,Ł_12_[*hz*^′^(*t*);*h*])^*T*^ is a linear difference operator.

Assuming that *z*(*t*) is sufficiently differentiable, we can expand the terms in (4) as a Taylor series about the point *t* to obtain the expression for the local truncation error
12

where the constant coefficients *C*_*q*_ = (*C*_1,*q*_,*C*_2,*q*_,…,*C*_12,*q*_)^*T*^, *q* = 0,1,… are given as follows:


#### **Definition1**

Let *p*_*j*_,*pj*′,*j* = 1,…,6 be positive integers, then, the block method (10) has algebraic order *p* = *min*{*p*_1_,…,*p*_6_,*p*1′,…,*p*6′}, *p*>1, provided there exists a corresponding constant *C*_*p* + 2_ such that the Local Truncation Error *E*_*μ*_ satisfies


where ∥·∥ is the maximum norm.

#### **Definition2**.

The block method (10) is said to be consistent if it has order at least one.

The block method (10) has order and error constant given by the vector *p*=6 and .

### Linear stability of the SOLMM

The linear-stability of the SOLMM is discussed by applying the method to the test equation *y*^′′^ = *λy*, where *λ* is expected to run through the (negative) eigenvalues of the Jacobian matrix  (see Sommeijer (1993)). Letting *q* = *λh*^2^, it is easily shown that the application of (10) to the test equation yields
13

where the matrix *M*(*q*) is the amplification matrix which determines the stability of the method.

#### **Definition3**.

The interval [ -*q*_0_,0] is the stability interval, if in this interval *ρ*(*q*)≤1, where *ρ*(*q*) is the spectral radius of *M*(*q*) and *q*_0_ is the stability boundary (see Sommeijer (1993)).

#### **Remark1**

We found that *ρ*(*q*)≤1 if *qε* [-4.552,0], hence, for the SOLMM, *q*_0_ = 4.552.

## Implementation of the SOLMM

The SOLMM was implemented in both block mode and predictor-corrector mode using a written code in PERL programming language and executed on a laptop computer with AMD Quad-Core A10-4600M Processor, 8GB of RAM and Windows 8.1 OS. The total program running time was acceptable, as shown in Tables 1, 2 and 3. The computational time complexity and space complexity of the algorithms for both modes of SOLMM used for the examples in this paper are polynomial. Details of the block mode implementation is given in Jator (2012) and the predictor-corrector implementation is discussed next.

**Table 1:** **Results, with** ***t*** ***ε*** **[ 0,1], for Example 1**

	PC-mode			Block-mode		
N	NFEs	Max error	CPU time	NFEs	Max error	CPU time
6	7	3.14×10^-3^	2.88×10^-2^	7	3.14×10^-3^	3.42×10^-2^
12	17	4.01×10^-5^	3.84×10^-2^	13	1.40×10^-5^	6.32×10^-2^
24	43	9.68×10^-7^	6.04×10^-2^	25	5.07×10^-8^	1.26×10^-1^
48	91	2.47×10^-5^	5.27×10^-2^	49	1.92×10^-10^	1.31×10^-1^
96	187	8.82×10^3^	9.09×10^-2^	97	5.31×10^-12^	2.64×10^-1^

**Table 2:** **Results, with** ***t*** ***ε*** **[1,8], for Example**2

	PC-mode			Block-mode		
N	NFEs	Max error	CPU time	NFEs	Max error	CPU time
6	7	2.40×10^-3^	2.11×10^-2^	7	2.240×10^-3^	1.47×10^-2^
12	17	9.23×10^-4^	1.18×10^-2^	13	2.42×10^-4^	4.13×10^-2^
24	43	1.51×10^-2^	2.13×10^-2^	25	1.23×10^-5^	3.37×10^-2^
48	91	8.78×10^0^	4.05×10^-2^	49	2.33×10^-7^	9.35×10^-2^
96	187	2.66×10^8^	7.70×10^-2^	97	1.79×10^-9^	1.48×10^-1^

**Table 3:** **Results, with**  **, for Example 3**

	PC-mode			Block-mode		
N	NFEs	Max error	CPU time	NFEs	Max error	CPU time
180	710	2.34×10^33^	6.84×10^-1^	362	1.95×10^-2^	6.82×10^1^
360	1430	5.81×10^59^	7.84×10^-1^	722	2.13×10^-4^	5.36×10^1^
720	2870	5.41×10^123^	1.15×10^0^	1442	8.30×10^-7^	8.46×10^1^
1440	5750	8.62×10^257^	1.93×10^0^	2882	3.40×10^-9^	1.24×10^2^
2880	11510	1.68×10^304^	3.12×10^0^	5762	1.38×10^-11^	2.46×10^2^

### Predictor-corrector mode algorithm

The initial block was used to start predictor-corrector algorithm, after which the predictor (14) and corrector (15) were used in a step-by-step fashion to provide the numerical solution from the second block to the end of the interval.

**Predictors.** The following predictors are derived via Theorem 2.1 by deleting the last row and column of matrix W.
14

**Correctors.** The last members of (8) and (9) are used as correctors.
15

## Numerical examples

### **Example1**.

We consider the IVP given by


### **Example2**.

We consider the given Bessel’s IVP solved on [ 1,8] (see Vigo-Aguiar and Ramos (2006)).




The theoretical solution at *t*=8 is .

### **Example3**.

We consider the nonlinear Fehlberg problem which was also solved in Sommeijer (1993).


### Comparison of block mode and predictor-corrector mode

The SOLMM is implemented in both predictor-corrector and block modes. The two approaches are compared by measuring their total number of function evaluations (NFEs) and CPU times in seconds. The block mode implementation is shown to be superior to the predictor-corrector mode implementation in terms of accuracy and the number of function evaluations. However, the predictor-corrector mode implementation uses less time than the block implementation. Details of the numerical examples are displayed in Tables 1, 2 and 3.

### Comparison of block method with other methods

The theoretical solution at *t* = 8 is . The errors in the solution were obtained at *t* = 8 using the SOLMM of order 7 and compared the the errors in (Vigo-Aguiar and Ramos 2006) which is based on the variable- step Falker method of order eight (VAR (8)) implemented in the predictor-corrector mode. The results given in Table 4 show that the SOLMM is more accurate than the method in (Vigo-Aguiar and Ramos 2006).

**Table 4:** **Absolute errors for Example**2

VAR (8)		SOLMM	
Steps	Errors	Steps	Errors
67	7.1122×10^-7^	60	2.49×10^-8^
82	9.2632×10^-8^	80	3.16×10^-9^
97	8.7834×10^-9^	100	6.04×10^-10^
112	1.2108×10^-10^	112	2.57×10^-10^

The maximum norm of the global error for the *y*-component is given in the form 10^-*CD*^, where CD denotes the the number correct decimal digits at the endpoint (see (Sommeijer 1993)). This problem has also been solved in (Sommeijer 1993) using the eighth-order, eight-stage RKN (H8) method constructed by Hairer (1977). We have chosen to compare this method of order 8 with our method of order 7, because the orders of the methods are very close. The results obtained using the *H*8 are reproduced in Table 5 and compared with the results given by our method. It is seen from Table 5 that our method performs better than those in (Sommeijer 1993) in terms of accuracy (smaller errors) and efficiency (smaller NFEs).

**Table 5:** **The correct decimal digit at the endpoint for Example 3**

H8		SOLMM	
NFEs	CD	NFEs	CD
400	0.3	362	1.7
800	2.6	722	3.7
1600	5.2	1442	6.1
3200	7.6	2882	8.5
6400	10.0	5762	10.9

## Conclusion

A SOLMM is proposed and implemented in both predictor-corrector and block modes. It is shown that the block mode algorithm is superior to the predictor-corrector mode algorithm in terms of accuracy and the number of function evaluations. However, the predictor-corrector mode implementation uses less time that the block implementation. the Details of the comparison of the numerical examples are displayed in Tables 1, 2, 3, 4 and 5. Our future research will be focus on developing a variable step version of the SOLMM in both modes.
